# Pharmacokinetic evaluation of quetiapine fumarate controlled release hybrid hydrogel: a healthier treatment of schizophrenia

**DOI:** 10.1080/10717544.2018.1458922

**Published:** 2018-04-12

**Authors:** Muhammad Akhlaq, Faiza Maryam, Abdelhamid Elaissari, Hashmat Ullah, Muhammad Adeel, Abid Hussain, Muhammad Ramzan, Obaid Ullah, Muhammad Zeeshan Danish, Shehla Iftikhar, Nighat Aziz

**Affiliations:** a Faculty of Pharmacy, Gomal University, Dera Ismail Khan, Pakistan;; b Univ Lyon, University Claude Bernard Lyon-1, CNRS, Lyon, France;; c Institute of Chemical Sciences, Gomal University, Dera Ismail Khan, Pakistan;; d Drug Regulatory Authority of Pakistan, Islamabad, Pakistan;; e Department of Pharmaceutics, University College of Pharmacy, University of Punjab, Lahore, Pakistan;; f Department of Oncology, SEENAR Hospital, Quetta, Pakistan;; g Department of Pharmacology, Gomal Medical College, Dera Ismail Khan, Pakistan;

**Keywords:** Quetiapine fumarate, cross-linking, hydrogels, pH-sensitive, pharmacokinetics

## Abstract

The current study aimed to rationally develop and characterize pH-sensitive controlled release hydrogels by graft polymerization of gelatin (Gel) and hydroxypropyl methyl cellulose (HPMC) in the presence of glutaraldehyde (GA) using quetiapine fumarate for the treatment of schizophrenia. The prepared hydrogels discs were subjected to various physicochemical studies including: swelling, diffusion, porosity, sol-gel analysis, Fourier transform infrared spectroscopy, differential scanning calorimetry, and scanning electron microscopy. Three different pH values (1.2, 6.8 and 7.4) were used to determine shape, transition, and controlled release behavior of prepared hydrogels. Various kinetic models including zero order, first order, Higuchi model and Power Law equation were applied on drug release data. The optimized hydrogels were subjected to *in vivo* studies using albino rabbits. Swelling and release results were found to be insignificant (*p* < .05) evidencing that there was no significant difference in swelling and drug release rate of hydrogels in different pH mediums. Swelling, porosity, gel-fraction, and drug released (%) were found to be dependent on concentrations of Gel, HPMC, and GA. Kinetic models revealed that QTP-F release followed non-Fickian diffusion. *In-vivo* studies contributed significantly higher plasma QTP-F concentration (*C*_max_), time for maximum plasma concentration (*T*_max_), area under the curve (AUC_0–inf_) and half-life (*t*_1/2_) as 18.32 ± 0.50 µg/ml, 8.00 ± 0.01 hrs, 6021.2 ± 5.09 µg.hrs/ml and 10.06 ± 0.43 hrs, respectively, for test-hydrogels when compared to reference market brand (Qusel^®^ 200 mg, Hilton Pharma, Karachi, Pakistan) QTP-F tablets. It might be concluded that QTP-F loaded pH-sensitive hydrogels were developed successfully with reduced dosing frequency for schizophrenia.

## Graphical abstract

**Figure:**
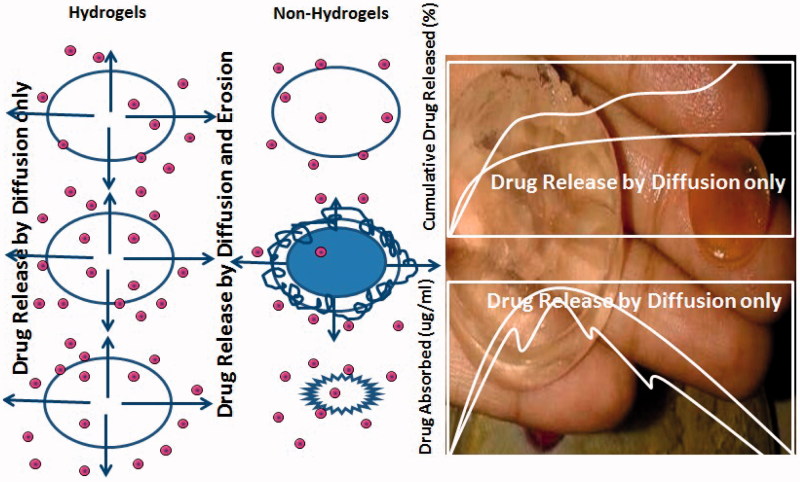

## Introduction

Polymer-based drug delivery systems containing swellable or nonswellable polymers are used to formulate a drug delivery device which releases the drug in a controlled manner (Mora et al., 2010). They are different from controlled released tablets. These formulations follow Zero Order kinetics (Howden, 2005). The rate of drug release could be predictable *in vivo* as well as *in vitro* (Barba et al., 2009). Oral route is the most convenient route for drug administration and immediate release tablets are the most commonly used type of dosage forms (Boyer et al., 2017). They are popular because of two main factors, that is, they can be produced on large scale which makes this dosage form economical while second factor is patient’s compliance because this dosage form is easy to use and provides therapeutic effects very effectively (Jeong et al., 2012). The main disadvantage of this dosage form is multiple dosing which requires monitoring for patients compliance (Carvalho & Mansur, 2017). Infrequent dosing leads to low concentration of drug in plasma which ultimately affects the therapeutic effect of pharmaceutical agent. All these factors are responsible for formulation of controlled release tablets, but the main drawback of controlled release tablets is dose dumping which leads to over dosing causing severe adverse effects (Biswal et al., 2016). To overcome this problem, polymer-based controlled release dosage form became the subject of interest for the researchers (Joner et al., 2008). A polymer that shows a sudden shift in its physical characteristics when its external environment changes is known as a stimuli-sensitive or smart polymer (García-Astrain et al., 2016). They are also known as intelligent polymers because small changes occur in response to an external trigger until a critical point is reached, and they have the ability to return to their original shape after trigger is removed (Gupta et al., 2002).

Quetiapine fumarate is an antipsychotic drug and is used in the treatment of schizophrenia, depression and anxiety disorders with a short half-life of about 7 hours that makes its dose 2 times a day (Ignácio et al., 2017). Schizophrenia is a severe mental disorder with very limited risk of life. It is characterized with hallucinations, cognitive deficits and delusions. QTP-F is a derivative of dibenzothiazepine and is classified as atypical antipsychotic drug. The enzyme responsible for its metabolism is cytochrome P450 3A4 which means that concurrent administration of any drugs that induce or inhibit this enzyme will affect QTP-F pharmacokinetics. Ingestion of food, gender, ethnicity, body weight and smoking has no effect on the bioavailability of this drug.

Quetiapine fumarate is available as immediate and extended release (ER) formulations. Extended release (ER) tablets of QTP-F were formulated to provide more convenient and simpler administrations for patients through once-daily dosing as compared to immediate release dosage forms (Mamo et al., 2008). Extended release formulation allows QTP-F concentration to be maintained at constant level for a constant period of time (Bandelow et al., 2010). The ER QTP-F formulations were also developed with the aim of reducing overdosing, thus minimizing side effects due to over-dosage and to improve compliance in patients with schizophrenia who normally skip doses because of cognitive problems (Osborne et al., 2016). STP-F sustained release matrix tablets were developed using natural polymer Gulmohar gum to achieve optimum therapeutic response, prolonged efficacy and decrease toxicity (Krishnaraj et al., 2012).

In the present investigation, quetiapine fumarate was employed in the controlled drug delivery system for extending the drug release for a prolonged period of time. Quetiapine fumarate controlled release hydrogels were prepared by graft polymerization of Gel and HPMC in the presence of glutaraldehyde. No previous work has been done on the reported formulation. QTP-F controlled release oral hydrogels were formulated to provide more convenient and simpler administrations for patients through once daily dosing as compared to immediate release tablets. This may allow drug to be released by diffusion mechanism thereby maintaining at constant levels for a desired time period. Formulations were prepared (*N* = 9) containing different concentrations of polymers and cross linker and were assessed for physical properties and *in-vitro* QTP-F release profiles. The optimized formulation was then compared with the commercially available oral QTP-F tablets (Qusel^®^ 200 mg, Hilton Pharma, Pk). The developed Gel/HPMC hydrogel might be advantageous in the sense that (a) the drug may be released in controlled manner following zero order kinetics thus reducing the dosing frequency and making it suitable for elderly patients with dementia and schizophrenia as they are difficult to deal with because of their mental condition. (b) It might reduce the chances of overdosing due to erosion of the polymer matrix. (c) Hybrid hydrogel might improve the mechanical strength of the gel thus decreasing the chances of erosion and abrupt drug release. (d) Graft polymerization technique might minimize the chances of dose dumping by packing the polymers tightly. (e) *In situ* drug-loading method might make this formulation suitable for water-insoluble drug like QTP-F.

## Materials and methods

### Materials

Quetiapine fumarate (QTP-F) was received as a gift sample from Olive Laboratories (Pvt. Ltd), Islamabad, Pakistan. Commercially available QTP-F tablets (Qusel^®^ 200 mg, Hilton Pharma, Karachi, Pakistan) was purchased from the local market, DIK, Pakistan. Gelatin and hydroxypropylmethylcellulose were used as monomer (Merck, Germany) and glutaraldehyde was used as a cross-linker (Merck, Germany), sodium hydroxide (Merck, Germany), potassium phosphate (monobasic), hydrochloric acid, acetonitrile (analytical grade), methanol (analytical grade), water (analytical grade) (Merck, Germany). All the chemicals were provided by Department of Pharmaceutics, Faculty of Pharmacy, Gomal University, DIK, KP, Pakistan.

### Methods

#### Preparation of gel/HPMC hybrid polymeric network

For the preparation of glutaraldehyde cross-linked Gel/HPMC hydrogels, two solutions (Aand B) were prepared (Caccavo et al., 2015; Cross et al., 2015). For preparation of solution ‘A’, distilled water (30 ml) was taken in a beaker (100 ml) and placed on a magnetic stirrer. When the temperature reached at about 40–45 °C, a specified quantities of gelatin (10.8, 14.4 and 18.5 g) were added to the distilled water and was stirred at 150–200 rpm. The solution was mixed for about 20 min until a clear solution was formed. After the complete dissolution of Gel, the beaker was removed from the stirrer and placed aside. The solution was then transferred to a test tube (10 ml). For preparation of solution ‘B’, distilled water (10 ml) was taken in a beaker and was heated to 80–90 °C. The specified quantity of HPMC (1.1, 0.8, 1.02 and 1.5 g) was added with constant stirring at 150–200 rpm for about 10 min until the polymer and water were evenly mixed. The solution was then transferred to a test tube. After formation of the two solutions, solution ‘A’ was added into solution ‘B’ with constant stirring on magnetic stirrer at 100–150 rpm for 15 min until homogenous solution was formed. Subsequently, after mixing of the solutions ‘A’ and ‘B’, specified quantities (1.5, 2, 3.5 and 4.6%) of GA were added in the solution dropwise with constant stirring. Volume was made up to 100 ml with distilled water. The solution was added in the test tubes and was allowed to congeal for three days at 25 °C. After the gel was formed, hydrogels were removed from the test tubes and cut into a size (5 mm) with the help of a sharp blade. The discs were placed in petri dishes and allowed to dry for three days and then re-dried in oven at 25 °C for 4 hours.

#### Evaluation of hydrogel

The hydrogel discs, before loading QTP-F, were subjected to a number of tests including swelling (dynamic and equilibrium), diffusion coefficient, sol-gel analysis and porosity.

#### Dynamic and equilibrium swelling studies

The pH of the medium interacts with the polymers (hydrophilic/hydrophobic) and activates the swelling behavior, allowing the release of the drug from the polymer matrix. Hence to find out the most suitable solvent (pH) having maximum effect on the swelling of hydrogels, the dried discs were weighed and immersed in HCl (0.1 M) pH 1.2 and in phosphate buffer solutions of pH 6.8 and 7.4 maintained at 37 ± 0.5 °C (Minhas et al., 2013). Samples were cleaned with tissue paper to remove extra solution and weighed after regular intervals of time (0.5, 1, 1.5, 2, 3, 4, 5, 6, 7, 8 hrs). After weighing the samples were placed back in the solution. Variation in weight is the indicative of quantity of water taken up by hydrogels after regular intervals of time (Carvalho & Mansur, 2017), dynamic swelling ratio was calculated using Equation (1).

(1) $$q=\frac{W_{\text{t}}}{W_{\text{d}}}$$

where *W*_t_ is weight of swollen gel at time *t* and *W*_d_ is weight of dry gel before swelling.

On completion of dynamic swelling studies, same samples were allowed to swell continuously until a constant weight was achieved (García-Astrain et al., 2016), equilibrium swelling ratio was calculated using Equation (2).

(2) $$q_{\left(\mathrm{Eq}\right)}=\frac{W_{\text{h}}}{W_{\text{d}}}$$

where *W*_h_ represents the weight of swollen gel at equilibrium and *W*_d_ represents the weight of dry gel before swelling.

#### Sol-gel analysis

Sol is the precursor for the production of gel. This test is performed to determine the percentage of sol fraction and gel fraction present in the hydrogel (Umejima et al., 1993). The dried unwashed hydrogels were weighed. Sol-gel analysis was performed by soxhelt extraction (Pyrex-A, Sigma-Aldrich, USA) using de-ionized water in especial apparatus at 80 °C for 4 hours. After extraction of uncross-linked polymers, the discs were removed and were dried at room temperature. After drying, discs were weighed again. All experiments were carried out in triplicates. Sol fraction and gel fraction of each disc were calculated using Equations (3 and 4).

(3) Sol fraction (%)=[Wo-W1Wo]×100

(4) Gel fraction (%)=100 – Sol fraction

where *W*_o_ represents the initial weight of the dry gel and *W*_1_ is the weight of dry gel after extraction (Gupta et al., 2014).

#### Porosity measurement

Porosity is the most important factor that affects the swelling of hydrogel. Porosity depends on the amount of cross-linker and the concentration of polymers used. Solvent replacement method was used to estimate the porosity of Gel/HPMC hydrogels. First initial weight was calculated and then the discs were placed in absolute ethanol for 12 hours. The discs were then removed from ethanol and final weight was recorded after cleaning with tissue paper. All experiments were carried out in triplicates and porosity was calculated using Equation (5).

(5) $$\mathrm{Porosity}=\frac{\left(M_{2}-M_{1}\right)}{\rho V}\times 100$$

where *M*_1_ and *M*_2_ represent the mass of hydrogel disc before and after dipping in ethanol respectively, ρ represents the density of absolute ethanol and *V* is the hydrogel volume (García-Astrain et al., 2016). 

#### *In situ* drug-loading method

Formulations showing optimum swelling ratios were re-formulated precisely and were loaded with QTP-F. Just after addition of cross-linker in the mixed solutions, specified quantity (2.00 g) of QTP-F was added. Solution was poured in to glass test tubes and was allowed to congeal for next three days. The hydrogels were taken out from the test tubes, cut, dried and re-dried in oven at 25 °C for 4 hours (Caccavo et al., 2015).

#### Drug content analysis

Two tests were performed to determine the amount of QTP-F loaded in the hydrogels. Three hydrogels were selected from each sample and the mean was calculated.

#### By extraction

This test was performed to determine the amount of drug loaded in each hydrogel disc. The drug-loaded hydrogel discs were placed in the buffer solution (pH 1.2) in which it showed maximum swelling for 24 hours. The buffer solution was filtered and drug content was determined using a manual HPLC system (Perkin Elmer, Series 200, Waltham, MA, USA) at 294 nm (Mirzaei et al., 2012).

#### Drug release studies

The release of QTP-F from hydrogel discs was determined (Kim et al., 1992) using USP dissolution apparatus type-II (paddle method) (PharmaTest, Hamburg, Germany) and HPLC system (Perkin Elmer, Series 200, Waltham, MA, USA). Weighed hydrogels were placed in 500 ml buffer solutions of varying pH (1.2, 6.8 and 7.4 pH) at 37 °C for 12 hours. Paddles were rotated at 100 rpm. Sample (5 ml) was withdrawn at pre-determined time intervals (0.25, 0.50, 1, 2, 3, 4, 5, 6, 7, 8, 9, 10, 11, 12 hrs) and withdrawn solution was replaced with fresh buffer solution. The samples were filtered (filter membrane pore size 0.45 mm) and amount of QTP-F released was estimated using HPLC at 294 nm (Mirzaei et al., 2012). All the experiments were carried out in triplicates, mean ± standard deviation was calculated.

#### Characterization

Surface morphology of the test hydrogel was examined using SEM (SEM; Joel JSM-5910, Japan) (Akhlaq et al., 2017). Fourier transform infrared spectroscopic (Mansur et al., 2008) and differential scanning calorimetric (Hataleyema et al., 1984) studies were conducted for QTP-F, gelatin, HPMC and hydrogels (loaded and unloaded).

#### *In vivo* evaluation

*In vivo* study decorum was accepted by the Ethical Committee (Ref No. 14/ERB/GU, dated 31/10/2016), Faculty of Pharmacy, Gomal University, DIK, KP, Pakistan. The approved procedure for handling the laboratory animals was tracked according to Act 1986. The basic pharmacokinetic constraints of reference QTP-F sustained release tablets (Qusel^®^ 200 mg, Hilton Pharma, Pk) and selected hydrogels were assessed using two groups of rabbits (Parallel-Design) each group covering 12 rabbits. Indigenous class of albino rabbits (both male and female rabbits, each weighing 3 ± 0.5 kg) was selected for the study. The selected breeds of rabbits were distributed into 2 clusters (each one containing 12 animals) and were retained on fasting for 24 hours prior to taking of test hydrogels and reference tablets and were kept for 24 hours more after postadministration. The test hydrogels and reference tablets were given using syringe (3 ml) with its barrel cut at the needle end. Water was permitted *ad libitum*. The standard food ‘white fish meat (10%), middling (18%), grass meal (20%) and brain (40%)’ was given to the rabbits 3 days before staring the experiments. For administration of QTP-F test-hydrogels and reference-tablets to the animals, a 3-ml syringe, with its needle end uniformly cut, was used. Tap water was given to rabbits u sing syringe (10 ml) built-in with oral tube to simulate with the human drug dosing. Centrifuged tubes (already holding sodium heparin) were used to accumulate the blood samples at predetermined time intervals (0 time before closing, 0.5, 1, 1.5, 2, 3, 4, 5, 6, 8, 10, 12, 18, 24, 36, 42 and 48 hours after closing) using cannula positioned in marginal ear vein. Samples were centrifuged (for 15 min, at 4000 revolution per min) and was reserved frozen (−20 °C) for further analysis.

#### Drug extraction from rabbit plasma

A single step (liquid-liquid extraction) technique was adopted for withdrawal of QTP-F from plasma using a published method with slight modification (Akhlaq et al., 2017). Plasma (500 µl) holding drug was obtained in a screw capped glass-tube and a mixture of hexane: ether (4 ml in 4:1 v/v) was added to it as extracting solvent. Then, the equal volume of the plasma (500 µl) was taken, extracted with diethyl ether (4 ml) and vortexed (vortex mixer, Gyromixer, Pakistan) for 5 minutes. The samples were then centrifuged (Kubota, Japan) at 3500 rpm for 15 min. The upper layer of the solvent was shifted into a vial (React Vial, Germany). The organic solvents were vaporized completely using nitrogen gas atmosphere at 40 °C. The subsequent deposits obtained in react-vial were re-formed using the same mobile phases (100 µl) for QTP-F.

#### Analysis of QTP-F concentration in rabbit plasma

An HPLC technique was adopted to analyze QTP-F samples according to a reported technique with small adjustment (Akhlaq et al., 2017). A manual HPLC system (Perkin Elmer, Series 200, Waltham, MA, USA) fitted with binary pump, UV detector, an integrator NCl-900, a degasser and a TCNa software was used. The collected plasma (20 µl) samples were inserted using syringe (50 µl) in injection port (Rheodyne). Quetiapine was identified at *ʎ*_max_ 294 nm using a reverse phase stainless steel (C 18, ODS Hypersil, 4.6 × 250 mm, 5 µm) analytical column (Thermo Electron Corporation, Waltham, MA, USA) fitted with guard-column. The filtered solvent (0.45 µl membrane filter; Sartorius, Germany) was degassed using a sonicator (Elma-D 78224, Germany) at pH 7.4. Acetonitrile and phosphate buffer solution (in ratio of 60:40 *v/v*) was cycled as mobile phase. The samples were analyzed (isocratically) at 1.0 ml/min stream rate. The quantification was completed using standard regression equation derived from standard curve (*r* ^2^ = 0.999).

#### Pharmacokinetic analysis and IVIVC

The pharmacokinetic constraints *t*_½_, *T*_max_, *C*_max_, AUC_0_, AUC_0-inf,_ MRT_0-48 hrs_ and Cl were calculated from the plasma concentration time profile of Quetiapine Fumarate reference tablets and test hydrogel using Kinetics Version-5 (Akhlaq et al., 2017).

The *in vitro* and *in vivo* correlation (IVIVC) was calculated by drawing Quetiapine Fumarate absorbed (%*F*_a_) against Quetiapine Fumarate released (%*F*_r_). The Quetiapine released (%) was taken from the dissolution data while Quetiapine Fumarate absorbed (%) was obtained by applying Wagner-Nelson model (Wagner & Nelson 1964).

(6) $$F_{\text{a}}=\frac{C_{\text{t}}+K_{\mathrm{el}}\times ({\mathrm{AUC}}_{\text{0}\text{-}\text{t}})}{K_{\mathrm{el}}\times ({\mathrm{AUC}}_{\text{0}\text{-}\infty })}\times 100$$

where *F*_a_ is the amount of quetiapine fumarate absorbed, *C*_t_ is the plasma quetiapine concentration at time *t*, *K*_el_ is the total elimination rate constant, AUC_0–t_ is the area under the curve (from time (0–t) and AUC_0–∞_ is the area under the curve (from time t–α).

### Statistical analysis

Statistical analysis was done using SPSS version 20. ANOVA, Student’s *t*-test was applied, a *p* value <.01 was considered as significant difference. The data were collected in triplicate and expressed as mean and standard deviation.

## Results

### Swelling profile

Results of dynamic and equilibrium swelling were found insignificant (*p* < .05) in the sense that Gel/HPMC hydrogels showed swelling both in acidic and basic mediums, but with different affinities for the different pH solvents.

**Figure 1.**
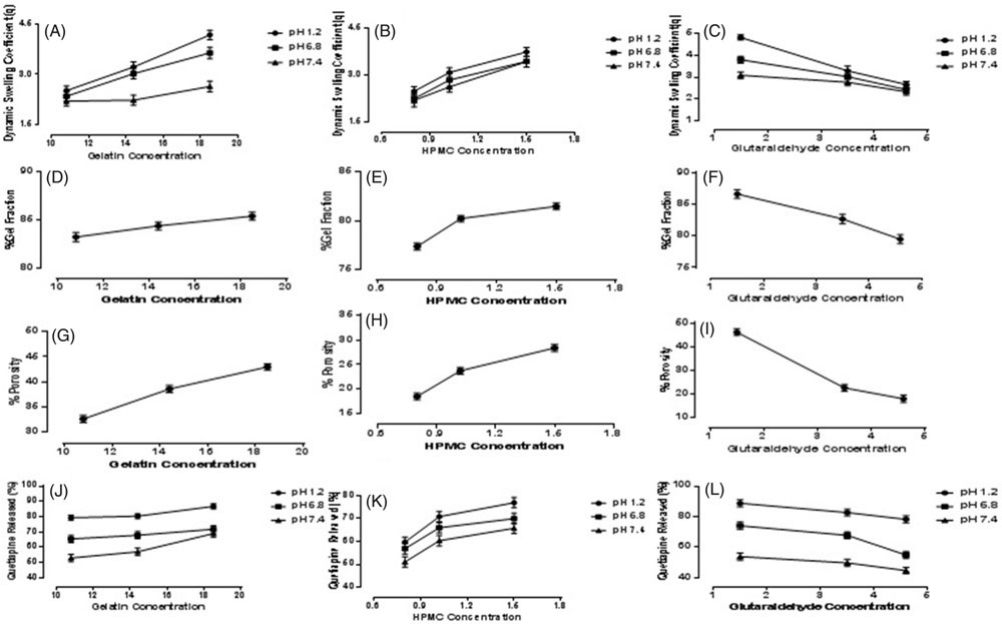
Dynamic swelling of Gel/HPMC with increasing concentration of (A) gelatin (B) HPMC (C) glutaraldehyde, gel fraction of Gel/HPMC with increasing concentration of (D) gelatin (E) HPMC (F) glutaraldehyde, porosity of Gel/HPMC with increasing concentration of (G) gelatin (H) HPMC (I) glutaraldehyde, QTP-F released of Gel/HPMC with increasing concentration Of (J) gelatin (K) HPMC (L) glutaraldehyde.

**Figure 2.**
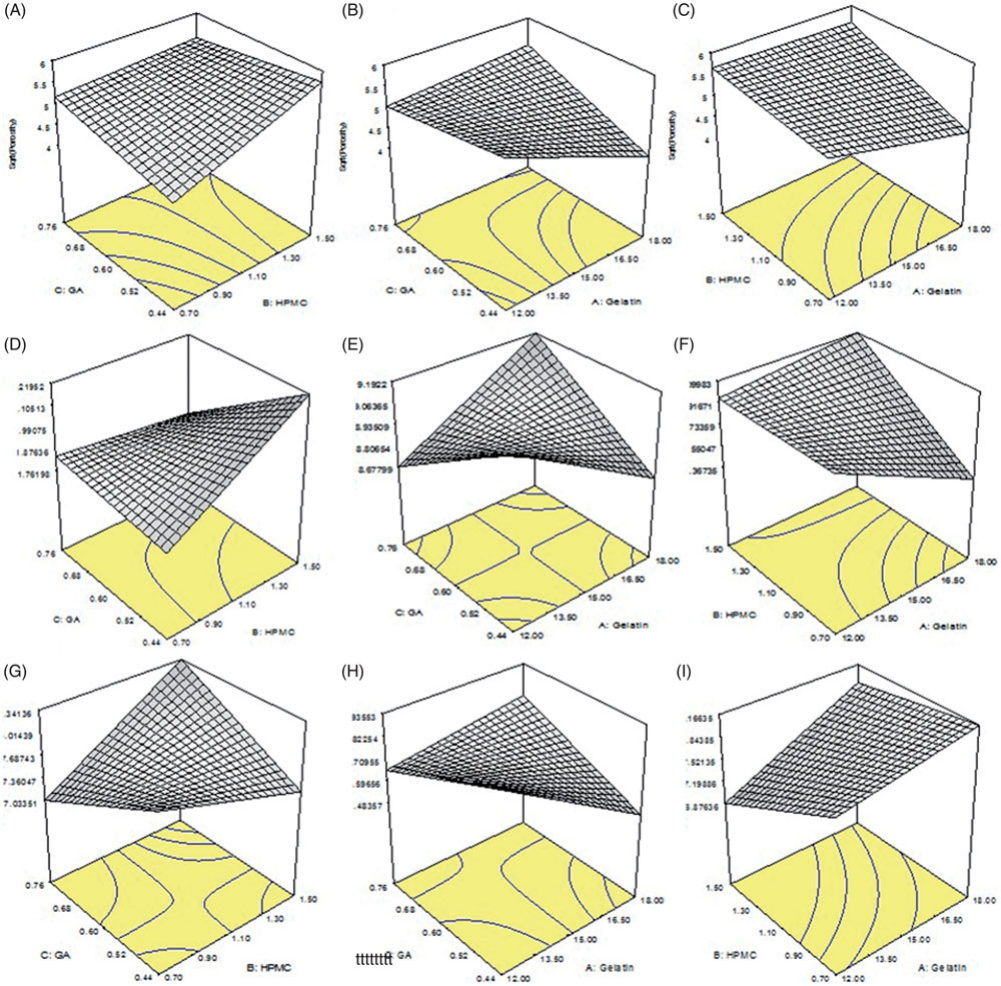
(A) 94.38/5.61 of Gel/HPMC and 2% GA (B) 90.56/9.43 of Gel/HPMC and 2% GA (C) 92.90/7.09 of Gel/HPMC and 1.5% GA showing porosity (D) 94.38/5.61 of Gel/HPMC and 2% GA (E) 90.56/9.43 of Gel/HPMC and 2% GA (F) 92.90/7.09 of Gel/HPMC and 1.5% GA showing 3-D structures dynamic swelling at pH 1.2 (G) 94.38/5.61 of Gel/HPMC and 2% GA (H) 90.56/9.43 of Gel/HPMC and 2% GA (I) 92.90/7.09 of Gel/HPMC and 1.5% GA showing 3-D structures dynamic swelling at pH 6.8.

Samples S_1_, S_2_, and S_3_ with different concentrations of gelatin (10.8, 14.4 and 18.5 g), keeping the concentrations of HPMC and GA constant, were evaluated to determine the effect of amount of Gel on swelling ratio. Figure 1(A) shows, as the amount of Gel was increased, the swelling of the samples was also increased and vice versa. Sample S_3_ showed maximum dynamic swelling 7.93 ± 0.1 at pH 1.2, while it was found to be 2.52 ± 0.30 almost minimum at pH 7.4. The maximum equilibrium swelling was observed to be 12.51 ± 0.2 at pH 1.2, while almost smaller to be 9.95 ± 0.56 for *S*_1_. Minimum dynamic swelling was observed to be 5.73 ± 0.5, while equilibrium swelling to be 9.51 ± 0.7 at pH 7.4 for *S*_2_. Figure 2(D) and (G) represents 3-D structures of Gel/HPMC hydrogel having Gel as actual factor showing swelling at pH 1.2 and 6.8.

Samples *S*_4_ to *S*_6_, varying concentrations of HPMC (0.8, 1.02 and 1.5 g) were used, keeping the quantity of Gel and GA constant to investigate the impact of HPMC on the samples. The samples showed a change in shape as the concentration of the HPMC was increased and vice versa. Figure 1(B) shows the effect of concentration of HPMC on the swelling behavior of hydrogels. *S*_6_ showed maximum dynamic swelling to be 7.83 ± 0.5 at pH 1.2 while maximum equilibrium swelling to be 12.01 ± 0.4 at 6.8 pH, sample *S*_4_ showed lowest dynamic swelling ratio to be 5.51 ± 0.5 at 7.4 pH and lowest equilibrium swelling ratio to be 9.01 ± 0.6 for *S*_5_ at pH 7.4. A 3-D structure, of Gel/HPMC hydrogel having HPMC as actual factor for swelling at pH 1.2 and pH 6.8, is shown in Figure 2(E) and (H), respectively.

Samples *S*_7_ to *S*_9_, varying concentrations (1.5, 3.5 and 4.6%) of GA were used, keeping the quantity of Gel/HPMC constant to investigate the effect of cross linker on swelling behavior of hydrogels. Figure 1(C) shows the impact of GA on the swelling behavior of hydrogels. Maximum dynamic swelling was shown by *S*_7_ 8.1 ± 0.5 at pH 1.2, while maximum equilibrium swelling was also shown by *S*_7_ to be 12.91 ± 0.5 at pH 1.2. Minimum dynamic swelling was observed for sample *S*_9_ to be 4.23 ± 0.7 at pH 7.4 and minimum equilibrium swelling was also shown by S_9_ to be 9.03 ± 0.2 at pH 7.4. Figure 2(F) and (I) represents the 3-D swelling of Gel/HPMC hydrogel having GA as an actual factor.

The Highest dynamic swelling ratio was observed to be 8.1 ± 0.2 for sample *S*_7_ and the highest equilibrium swelling ratio to be 12.91 ± 0.8 for sample *S*_7_ at pH 1.2 While dynamic swelling ratio was found to be 4.23 ± 0.5 for sample *S*_9_ at pH 7.4 and equilibrium swelling ratio was found to be 9.01 ± 0.7 for *S*_5_ at pH 7.4. Sample *S*_7_ showed significantly controlled release of QTP-F (*p* < .01).

### Sol-gel analysis

Sol-gel analysis was performed to determine the amount of uncrossed fraction of polymer in hydrogel. The results revealed that the gel fraction of hydrogels also depend on the quantity of Gel, HPMC and GA. The sol-gel was found to be 82.89 ± 1.21% for *S*_1_, 84.09 ± 1.43% for *S*_2_, 85.09 ± 1.32% for *S*_3_, 77.09 ± 1.54% for *S*_4_, 79.99 ± 1.34% for *S*_5_, 81.23 ± 1.55% for *S*_6_, 86.32 ± 1.34% for *S*_7_, 82.13 ± 1.54% for *S*_8_ and 78.98 ± 1.65% for *S*_9_.

Overall, maximum sol-gel fraction was observed in *S*_7_, second highest was observed in *S*_3_ and least was observed in *S*_9_ which were calculated to be 86.32 ± 1.34%, 85.09 ± 1.32% and 78.98 ± 1.65% respectively. Figure 1(D) shows that as the concentration of Gel was increased sol-gel fraction also increased. Sample *S*_3_ showed maximum percentage of about 85.09 ± 1.32%, second highest value was calculated to be 84.09 ± 1.43% in *S*_2_ while the least to be 82.89 ± 1.21 was observed in *S*_1_. Increasing the concentration of HPMC, increased the percentage of sol-gel as is shown in Figure 1(E). Highest value was calculated to be 81.23 ± 1.55% in *S*_6_, second highest value was 79.99 ± 1.34% in *S*_5_ and lowest was 77.09 ± 1.54% in *S*_4_. Figure 1(F) reveals that increasing the concentration of GA decreased the sol-gel fraction of hydrogels. The maximum percentage of sol-gel was calculated to 86.32 ± 1.34% in *S*_7_, next was about 82.13 ± 1.54% in *S*_8_ and 78.98 ± 1.65% in *S*_9_ on the basis of increasing concentration of cross linker.

### Porosity measurement

To investigate the effect of polymer and cross linker on porosity of hydrogel, various formulations of hydrogels were developed with varying concentrations of Gel, HPMC and GA. Porosity was observed to be 32.13 ± 1.54% for *S*_1_, 38.09 ± 1.43% for *S*_2_, 42.54 ± 1.51% for *S*_3_, 18.03 ± 1.32% for *S*_4_, 23.24 ± 1.66% for *S*_5_, 27.90 ± 1.71% for *S*_6_, 45.12 ± 1.88% for *S*_7_, 21.56 ± 1.59% for *S*_8_ and 16.90 ± 1.76% for *S*_9_.

**Figure 3.**
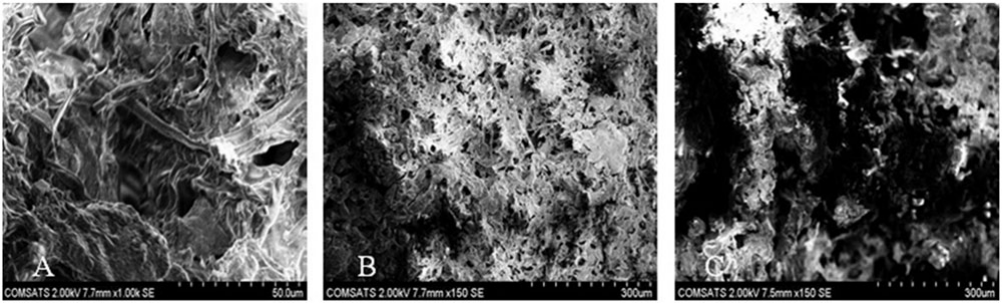
Legend surface morphology of Gel/HPMC hydrogel formulations (A) *S*_3_ (B) *S*_6_ (C) *S*_7_.

It was observed that samples (*S*_1_ to *S*_3_) showed increase in porosity with increasing concentration of Gel as shown in Figure 1(G). According to gelatin concentration, *S*_3_ with maximum quantity of gelatin showed maximum porosity of 2.54 ± 1.51%, second highest percentage of porosity was seen in S_2_ approximately 38.09 ± 1.43% and the least was observed in *S*_1_ to be 32.13 ± 1.54%. Effect of gelatin on porosity can be seen in 3-D graph Figure 2(A). Figure 3(A) shows the pores seen in the surface of sample *S*_3_ hydrogel. On the other hand, sample (*S*_1_ to *S*_3_) showed increased porosity with increasing concentration of HPMC as shown in Figure 1(H). The sample with highest amount of HPMC showed maximum porosity calculated to be 27.90 ± 1.71% in *S*_6_, the second highest porosity in *S*_5_ to be 23.24 ± 1.66% while the least porosity was observed in *S*_4_ to be 18.03 ± 1.32%. Figure 2(B) represents the 3-D structure having HPMC is the actual factor. Figure 3(B) could also be in good agreement with Figure 2(B) regarding the porosity of sample *S*_6_. Samples (*S*_7_ to *S*_9_) showed decrease in porosity with increasing the concentration of GA as evidenced in Figure 1(I). The maximum percentage of porosity was observed to be 45.12 ± 1.88% in sample *S*_7_, second highest porosity was observed to be 21.56 ± 1.59% in S_8_ while the least amount of porosity was observed to be 16.90 ± 1.76% in *S*_9_. The 3-D structure (Figure 2(C)) shows the effect of GA on porosity also observed in Figure 3(C) in the surface of sample *S*_7_.

### Drug release profile

Drug release profile was established to investigate the amount of drug released from the hydrogels. Impact of pH and concentrations of Gel, HPMC and GA on the amount of drug release are discussed below.

**Figure 4.**
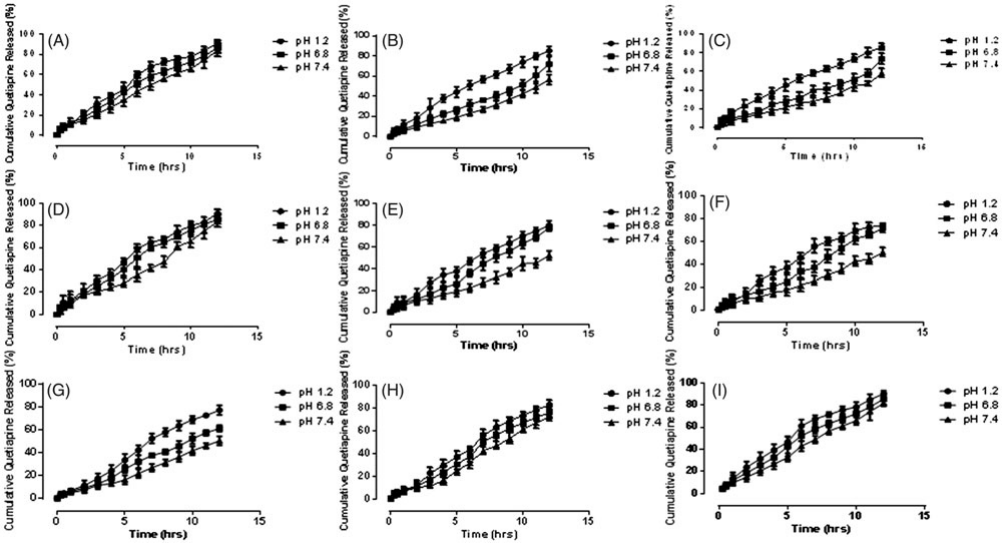
Cumulative % release of QTF-F from Gel/HPMC hydrogels (A) (90.75/9.24) using 2% of GA (B) (92.9/7.09) using 2% of GA (C) (94.38/5.61) using 2% of GA (D) (94.73/5.26) using 2% of GA (E) (93.38/6.61) using 2% of GA (F) (90.56/9.43) using 2% of GA (G) (90.56/9.43) using 1.5% of GA (H) (90.56/9.43) using 3.5% of GA (I) (90.56/9.43) using 4.6% of GA, after 12 hours at pH 1.2 (•), pH 6.8 (▪) and pH 7.4 (▲).

Samples *S*_1_, *S*_2_ and *S*_3_ (10.8 g, 14.4gs and 18.5 g) were selected to determine the effect of concentration of Gel on release of QTP-F from the samples. The amount of HPMC and GA was kept constant while that of Gel was increased gradually. The results showed that as the quantity of gelatin was increased, the amount of QTP-F release also increased and vice versa (Figures 1(J) and 4(A–C)). Sample *S*_3_ showed maximum drug release to be 83.12 ± 0.31% at pH 1.2 while sample S_1_ minimum to be 50.25 ± 0.22% at pH 7.4. Samples *S*_4_, *S*_5_ & *S*_6_ (0.8 g, 1.02 g and 1.5 g) were selected to determine the effect of concentration of HPMC on release of QTP-F from the hydrogels. Varying concentrations of HPMC were used while concentration of Gel and GA were kept constant. It was observed that higher is the concentration of HPMC, higher is the swelling and vice versa (Figures 1(B) and 4(D–F)). Sample *S*_6_ showed 84.34 ± 0.43% QTP-F released at pH 1.2 while *S*_4_ depicted 47.62 ± 0.14% at pH 7.4. Samples *S*_7_, *S*_8_ & *S*_9_ (1.5%, 3.5% and 4.6%) were selected to determine the effect of concentration of GA on release of QTP-F from the hydrogels (Figures 1(C) and 4(G–I)). Sample *S*_7_ showed maximum amount of QTP-F to be 87.82 ± 0.92% released at pH 1.2 while sample *S*_9_ to be 47.65 ± 0.38% at pH 7.4. Maximum QTP-F release was observed at pH 1.2 and second highest release at pH 6.8 (see Figures 1(J–L) and 4(A–I). The maximum QTP-F release was observed to be 87.82 ± 0.92% at pH 1.2 by sample *S*_7_ and minimum to be 47.65 ± 0.38% at pH 7.4 by sample *S*_9_.

### Drug content analysis

QTP-F leaded to different samples of hydrogels was estimated using two different techniques; by weight and by extraction method. It was observed that QTP-F loaded to various hydrogels samples was found to be 0.181, 0.166, 0.222, 0.166, 0.200, 0.200, 0.181, 0.166, 0.222 grams to samples S_1_ to S_9_ respectively by weight method. While it was found to be 0.164, 0.144, 0.143, 0.188, 0.161, 0.125, 0.173, 0.164, 0.144 grams of QTP-F loaded to samples S_1_ to S_9_ respectively by extraction technique (no need to add figure).

### Fourier transform infrared spectroscopy (FT-IR)

**Figure 5.**
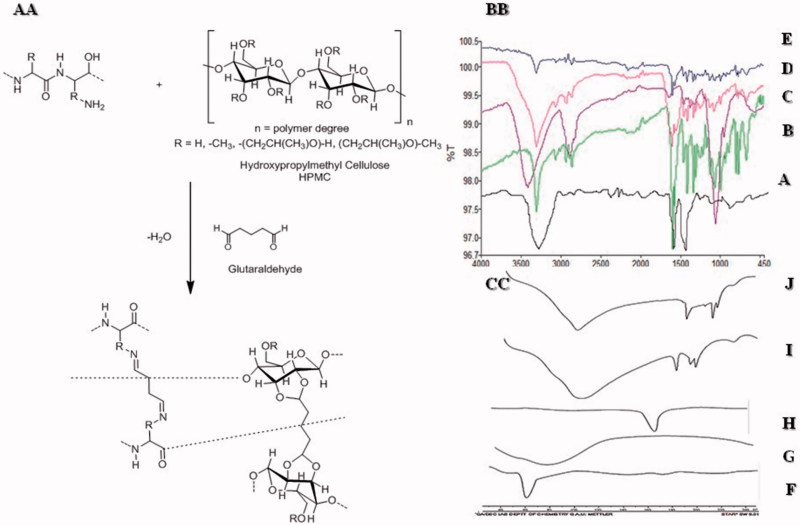
(AA) Synthesis of Gel/HPMC hydrogel (BB) FTIR of (A) Gelatin (B) QTP-F (C) HPMC (D) Unloaded hydrogel (E) Loaded hydrogel (CC) DSC of FTIR of (A) Gelatin (B) QTP-F (C) HPMC (D) Unloaded hydrogel (E) Loaded hydrogel.

The FT-IR absorption range and band task of gelatin for specific groups was portrayed (Yin et al., 2009). The absorption bands at 3260.73 cm^−1^ showed (N-H stretching), 1627 cm^−1^ peak (amide I, C–N stretching and C=O), 1536.50 cm^−1^ (amide-II) while 1241 cm^−1^ (amide-III) can be assigned to the distinctive bands of gelatin. Finally, the skeletal stretching arises at 665.60 cm-1 which showed N–H bond (Figure 5(BB–A)). Figure 5(BB–C) Represents the spectra of HPMC and the band at 3421.48 cm^−1^ was due to stretching of hydroxyl (OH) group, 2891.10 cm^−1^ represented C–H bond, 1456.16 cm^−1^ –CH_2_ group while 1377 cm^−1^ indicated the vibration of –OH group and finally at 1066.08 cm^−1^ C–O groups (Weerd & Kazarian, 2005). Figure 1(BB–B) shows peaks of QTP-F were observed at 3318 cm^−1^ representing -OH stretching, 3063 cm^−1^ C=H stretching in the two aromatic rings attached to thiazepine, 2862 and 2945 cm^−1^ showed –C–H stretching, 1597 cm^−1^ (carbonyl stretching of fumarate), 1064 cm^−1^ (–C–O–C– symmetrical stretching, 1404 cm^−1^ (aliphatic H bending), 1327 cm^−1^ (C–N stretching) 767 and 794 cm^−1^ (two distributed benzene ring) and 649 and 660 cm^−1^ (C–S–C stretching) (Figueroa et al., 2009). Figure 5(BB–D) depicts the absence of peak at 1700–1750 cm^−1^ showed stretching for 0 = C–H (glutaraldehyde) indicating strong chemical reaction between HPMC and glutaraldehyde. Gelatin formed shift base with GA due to which a broad peak of –NH_2_ at 3210 cm^−1^ was absent confirming the formation of shift base between GA and gelatin. It was evident from the FT-IR spectra of the hydrogels that there had been reduction in intensity of –OH stretching vibration peak (*n* = 3250 and 3495) as compared to usual -OH stretching peaks of HPMC and gelatin. This could be attributed to strong hydrogen bonding or strong intermolecular interaction between gelatin and HPMC. No chemical interaction between QTP-F and hydrogel was observed as evident in the IR spectra of QTP-F loaded hydrogel sample (Figure 5(BB–E)).

### Differential scanning calorimetry (DSC)

Differential scanning calorimetric studies were conducted for gelatin, HPMC, QTP-F, unloaded and loaded hydrogels. Thermogram of gelatin showed a sharp peak at 60 °C representing its characteristic melting point (Figures 5(CC–F)). Thermogram of HPMC depicted a sharp peak at 78 °C presenting its distinctive melting point (Figures 5(CC–G)). Figures 5(CC–H) symbolizes the thermogram of QTP-F with a sharp peak at 173 °C attributing to its specific melting point (Datto et al., 2009). The thermograms of gelatin and HPMC showed the endothermic peaks at their respective temperatures 50 °C and 78 °C, respectively. When the gelatin and HPMC were interacted chemically using glutaraldehyde as cross-linker, the endothermic broader peak was achieved at 100 °C while sharp peaks were located at 180 °C and 200 °C indicating interaction between Gel/HPMC in unloaded hydrogels. The results were also supported by FT-IR spectra indicating a sound chemical interaction between gelatin and HPMC cross-linked with glutaraldehyde (Figure 5(CC–I)). The thermogram of QTP-F-loaded sample showed that the endothermic peaks of the drug were shifted to 194 °C from 173 °C, indicating some sort of interaction between the drug and polymers but FT-IR spectra revealed that the interaction of drug and polymers might be physical not the chemical (Figure 5(CC–J)).

### Scanning electron microscopy (SEM)

Surface morphology of QTP-F loaded hydrogel was studied using SEM (Figure 3). The legend surface morphology of different formulations *S*_3_, *S*_6_ and *S*_7_ might suggest that there was a super-porous surface seen in all the hydrogel discs. The uniform distribution of the monomers in the hydrogel might lead to the inter-connected pores after gelling which might be clearly observed in the figure. The SEM images for all the samples portray inter-connected pores which actually contain numerous capillary channels in the polymer matrix. These capillary channels allow the water molecule to slowly diffuse into the polymer matrix and dissolve the QTP-F and then release out in sustained manner. Almost all the SEM images are showing the interconnected pores with narrow capillaries with significant difference in size depending upon the concentration of the polymers and the cross-linker, which might be better observed in case of porosity, swelling and QTP-F release studies.

### *In vivo* evaluation

**Figure 6.**
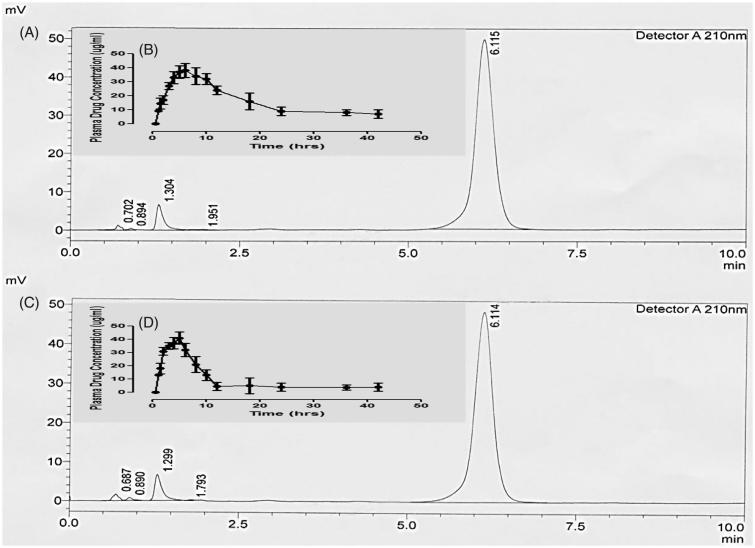
Sample HPLC chromatogram of QTP-F test hydrogel in plasma (A), Plasma drug concentration (µg/ml) profile of QTP-F test hydrogel *in-vivo* (B), Sample HPLC chromatogram of QTP-F reference tablet (Qusel^®^, Hilton Pharma, Karachi, Pakistan) in plasma (C), Plasma drug concentration (µg/ml) profile of QTP-F reference tablet (Qusel^®^, Hilton Pharma, Karachi, Pakistan).

Optimized QTP-F hydrogel formulation was selected for *in vivo* estimation after a comprehensive *in vitro* experimental study. Figure 6 designates characteristic chromatograms of standard QTP-F solution of rabbit plasma spiked after administration of test QTP-F hydrogels and the reference tablets. The *C*_max_ was obtained at 5.0 min (retention time). The mean absolute recovery of QTP-F, obtained from 6 aliquot samples, was found to be more than 95% over concentration range of 0.25–10 µg/ml. The standard calibration curve was found to be linear (*R* ^2^ = 0.999).

The mean plasma concentration–time contours of QTP-F test hydrogels and reference. The *in vivo* experiments both for plasma concentration of test hydrogels and reference tablets are insightful of comparatively extended-release rate of drug absorption. Conversely, it could be observed that more extended release rates might be obtained for test hydrogels (the drug remained in the body for 36 hours) equated to the reference tablets (the drug remained in the body for 12 hours).

Various pharmacokinetic considerations (based on the plasma level time curve) are accessible. The treatment effect was analyzed using a *two-tailed t*-test (SPSS 12.0) for comparing the results of test hydrogels with reference tablets. Mean half-life (*t*_1/2_) of QTP-F test hydrogels and reference tablets were originated to be 11.03 ± 0.12 to 6.42 ± 0.19 h (*p* < .01), respectively. Mean *C*_max_ was found to be as17.98 ± 0.93 and 17.24 ± 1.08 µg/ml (*p* < .05), Mean *T*_max_ for test hydrogels and reference tablets were found to be 2.20 ± 1.32 and 7.99 ± 0.02 hours (*p* < .05) hours correspondingly. Area under the curve AUC_0_ and AUC_0–inf_ for QTP-F test hydrogels and reference tablets were found to be 18.06 ± 1.03 and 17.1 ± 0.92 µg × hrs/ml and 5721.7 ± 7.11 and 5044.2 ± 4.11 µg × hours/ml (*p* < .05) respectively. Mean residence time for test hydrogels was found to be 13.11 ± 1.22 hours, while that for reference tablets to be 8.02 ± 0.43 hours (*p* < .01).

## Discussion

The shift of the IR spectra and thermogram peaks indicated that the gelatin containing amine (–NH_2_) group might have interacted with glutaraldehyde containing carbonyl group (H–C=O) while HPMC containing hydroxyl (–OH) group might have interacted with carbonyl group of Glutaraldehyde (H–C=O) (Speer et al., 1980). The chains formatted between Gel/GA and HPMC/GA interacted with each other through hydrogen bonding (FT-IR graph 5.82 and DSC graph 5.87). The drug loaded samples showed a shift in peak which might be due to physical interaction between the (–OH) groups of QTP-F and HPMC (Datto et al., 2009).

Swelling increases as the quantity of Gel increases. The functional groups of Gel responsible for swelling of hydrogels are amine (–NH_2_) and carboxylic acid group (–COOH) (Raafat, 2010). Amine contains basic nitrogen and is basically a derivative of ammonia with one or more hydrogen atom(s) replaced by alkyl or aryl group. At pH below pKb of basic components (acidic pH), ionization (protonation) occurs that results in formation of –NH_3_^+^ ions and due to the presence of similar cations, electrostatic repulsion takes place within them resulting in swelling of hydrogel in acidic medium (Gil et al., 2005). Gel also contains -COOH group which is an acidic component and at pH above pKa (basic medium) loses H + ion thus becomes COO^−^, ionization of COOH group at pH 7.4 causes the repulsion of anions due to electrostatic repulsion and thus swelling of the hydrogel occurs in basic medium. Because of the presence of these functional groups, Gel/HPMC hydrogel showed swelling at both acidic and basic pH (Lou & Chirila, 1999).

HPMC plays a major role in swelling of hydrogel because of its hydrophilicity and swellability nature. The reason for this nature of HPMC is due to the presence of hydroxypropyl contents of HPMC that has the ability to interact with water easily due to which the formulation has the ability to swell in the presence of water. But there was a shift in swelling towards basic medium as the concentration of HPMC increased because HPMC shows more swelling in basic medium which was also evident in its swelling behavior (Ranjha & Qureshi, 2014).

Lesser the amount of cross-linker, greater will be the swelling behavior and vice versa. The reason for this behavior is that the cross-linker forms physical entanglement between the polymers of hydrogel. The influence of increasing cross-linking can be described by decrease in mesh size of network (Atta & Abdel-Azim, 1998; Padhi et al., 2016). Three main reasons due to which amount of GA decreases the swelling behavior of hydrogel are (a) the amino group of polymers are mainly responsible for the swelling behavior and the cross-linker mainly conceals the amino group thus decreasing the ability of polymer to swell (b) the other reason is that higher cross-linking reduces the process of ionization which is also responsible for swelling of polymers of hydrogel (c) higher concentration of cross-linker decreases the relaxation of polymer chains resulting in lesser swelling of hydrogels (Mirzaei et al., 2012).

Maximum swelling (pH 1.2) was due to the presence of –NH_2_ group which is a basic component and is present in Gel while the quantity of HPMC was negligible as compared to the quantity of Gel so its effect was lesser as compared to Gel. On the other hand, the impact of cross-linker was much more evident in Gel/HPMC hydrogels as compared to both polymers because the cross-linker is basically responsible for the entanglement of the polymers and its varying quantity showed that it has a more dominant impact on all the characteristics of hydrogel including both dynamic and equilibrium swelling ratio, porosity, sol-gel fraction and drug release rate.

Sol-gel analysis is basically performed to determine the amount of uncrossed polymers. The results showed that as the quantity of polymers increased and cross-linker decreased, the percentage of uncrossed polymers increased because cross-linker links the two chains of polymers and if the quantity of polymer is higher than the amount of cross-linker then it will affect the amount of entanglement between the polymers leaving behind more quantity of uncrossed polymers (Jeong et al., 2012). 

Porosity is the number of pores present in the formulation and is a very important characteristic of hydrogel as both swelling and rate of drug release depend on it. Porosity is directly proportional to the quantity of polymers and inversely proportional to the amount of cross-linker but higher dependence is on the quantity of cross-linker because it is responsible for the linking of the two water soluble polymers creating a mesh like structure in the hydrogels. So, greater the amount of cross-linker, smaller will be the mesh size and vice versa. Hydrogels with smaller mesh size will swell less and in return lesser drug will be released. The more compacted hydrogel matrix with higher quantities of the cross-linker might result in the synthesis of more cross-linked polymers. The resulting polymeric hydrogel might not allow the water molecules to rush in faster inside the matrix and allowing the drug molecules to get dissolved and diffuse out easily (Ruggero et al., 1994; Raafat, 2010).

Gel/HPMC hydrogel follows the swelling controlled drug release mechanism. In this mechanism, the hydrogel changes its shape as it reaches the favorable environment. This shape transition depends on the amount of polymers (Gel and HPMC) and cross linker (GA) and degree of porosity (discussed above). The water enters inside the hydrogels through the pores as a result the dosage form swells up and releases the drug (Kim et al., 1992).

IVIVC is taken as a scientific exemplary to analyze the association among an *in vitro* performance parallel *in vivo* response. Characteristically the *in vitro* performance demonstrates the rate at which a drug is released from the dosage form while the *in vivo* response tells about the plasma drug concentration. Five levels of the relationship are given as level-A, level-B, level-C, level-D, and level-E for *in vitro in vivo* correlation. Among the five levels of correlation the level-A correlation describes the paramount class of correlation indicating point to point association between *in vitro* dissolution rate and *in vivo* absorption rate. Consequently, to establish IVIVC, segment of drug absorbed (%) (Fa, Y-axis) was plotted alongside fraction of drug released (%) (Fr, X-axis) (Wagner & Nelson, 1964). A good *in-vitro and in-vivo* correlation (*R* ^2^ = 988) indicating that the hydrogels were efficacious adequate for additional assessment. Nonetheless, the tablet of QTP-F exhibited fewer linearity with coefficient of determination (*R* ^2^) 0.876, while QTP-F test hydrogels presented a good linear relationship between the drug released *in-vitro* and drug absorbed *in-vivo* along with prolonged MRT_0-t_ and *t*_1/2_ values as compared to the reference tablets.

## Conclusions

In this study, controlled release Gel/HPMC hydrogels were developed in which GA was selected as a cross-linker and *in situ* drug-loading method was used. The developed hydrogels were pH sensitive with the ability to swell at different pH values. Different buffer solutions of pH 1.2, 6.8 and 7.4 were used to determine the swelling of the polymeric material. Swelling behavior showed a regular variation with different concentrations of Gel, HPMC and GA. Swelling behavior was directly proportional to the concentration of polymers (Gel and HPMC) and inversely proportional to the concentration of cross-linker (GA) which means that increasing concentration of Gel and HPMC increased the swelling of hydrogels and decreasing the quantity of GA increased the swelling behavior of hydrogels and vice versa. Another important factor is the amount of drug released from the hydrogel which also depended on the composition of the formulation and pH of the medium. The rate of drug release was faster at pH 1.2 then at 6.8 and lastly at 7.4. For this study, *In situ* drug-loading method was used because the drug used in the formulation is water insoluble so postloading method was difficult to apply. Characterizations like FT-IR and DSC confirmed that Gel/HPMC interacted with each other and hydrogel was formed. Significantly maximum amount of swelling and controlled drug release rate was exhibited by *S*_7_ containing the least quantity of GA about (1.5%). It was concluded that these hydrogels were pH sensitive as well as time dependent and have the ability to behave intelligently to their environment so they can be used as controlled release drug delivery system for water-insoluble drugs.
